# Risk Factors and Characteristics of Low Pathogenic Avian Influenza Virus Isolated from Commercial Poultry in Tunisia

**DOI:** 10.1371/journal.pone.0053524

**Published:** 2013-01-11

**Authors:** Wafa Tombari, Mathilde Paul, Jihene Bettaieb, Imen Larbi, Jihene Nsiri, Imen Elbehi, Latifa Gribaa, Abdeljelil Ghram

**Affiliations:** 1 University Tunis El Manar, Veterinary Microbiology Laboratory, Institut Pasteur de Tunis, Tunis- Belvédère, Tunisia; 2 INRA, UMR 1225 IHAP, Toulouse, France; 3 Universiy de Toulouse INP, Ecole Nationale Vétérinaire de Toulouse, Toulouse, France; 4 Medical Epidemiology Unit, Pasteur Institute of Tunis, Tunis, Tunisia; Centers for Disease Control and Prevention, United States of America

## Abstract

**Objective:**

Estimate the seroprevalence of influenza A virus in various commercial poultry farms and evaluate specific risk factors as well as analyze their genetic nature using molecular assays.

**Materials and Methods:**

This report summarizes the findings of a national survey realized from October 2010 to May 2011 on 800 flocks in 20 governorates. Serum samples were screened for the presence of specific influenza virus antibodies using cELISA test. Additionally, swab samples were tested by real time and conventional RT-PCR and compared with results obtained by others assays. Phylogenetic and genetic analyses of the glycoproteins were established for some strains.

**Results:**

Out of the 800 chicken and turkey flocks tested by cELISA, 223 showed positive anti-NP antibodies (28.7%, 95% CI: 25.6–32.1). Significantly higher seroprevalence was found among the coastal areas compared to inland and during the autumn and winter. Broiler flocks showed significantly lower seroprevalence than layers and broiler breeders. The influenza virus infection prevalence increased after the laying phase among layer flocks. In addition, AIV seropositivity was significantly associated with low biosecurity measures. The Ag EIA and rRT-PCR tests revealed significantly higher numbers of AI positive samples as compared to cell cultures or egg inoculation. All new strains were subtyped as H9N2 by real time and conventional RT-PCR. Drift mutations, addition or deletion of glycosylation sites were likely to have occurred in the HA and NA glycoproteins of Tunisian strains resulting in multiple new amino acid substitutions. This fact may reflect different evolutionary pressures affecting these glycoproteins. The role of these newly detected substitutions should be tested.

**Conclusion:**

Our findings highlight the potential risk of AIV to avian health. Strict enforcement of biosecurity measures and possible vaccination of all poultry flocks with continuous monitoring of poultry stations may ensure reduction of AIV prevalence and avoid emergence of more pathogenic strains.

## Introduction

Avian influenza (AI) is a respiratory disease. Its severity depends on many factors including host age, virus strain, and secondary infections. The causative agent is prevalent worldwide. Influenza A virus belongs to the family *Orthomyxoviridae* and the genus *Influenzavirus A*, and is characterized by a segmented, single-stranded, negative-sense RNA genome. This genus is subdivided into 17 hemagglutinin (HA) and 9 neuraminidase (NA) subtypes. AI virus infects domestic poultry, mammals and humans, and is thought to have originated in migratory wild birds [1].

Influenza A viruses are classified as either highly pathogenic AI (HPAIV), causing severe systemic disease with high mortality, or low pathogenic AI (LPAIV) inducing relatively mild clinical signs in broilers and drop in egg production in layers [2].

Recently, LPAIV H9N2 subtype has been isolated worldwide from different types of terrestrial poultry [3], [4]. Initially concentrated in Asia [5], outbreaks subsequently spread to Africa, the Middle East [6], [7], and America [8] causing significant economic losses related to increased mortality and decreased production in poultry industry [9]. It has also been reported that H9N2 avian influenza virus can cross species barrier and infect humans [10].

Monitoring AI viral infections in domestic and wild birds is therefore important to control animal diseases and prevent human pandemics. Many state laboratories participate in the surveillance of AI activity and contribute to the early recognition of newly emerging epidemic strains [11], [12].

Serological surveillance of antibodies against AIV is of great importance in preventing and controlling AI infection. Identification of both H and N subtypes is highly essential for epidemiological studies. Nowadays, the majority of field surveys of LPAIV are based on serological assays; molecular methods such as real-time reverse transcription PCR (rRT-PCR), which have been proven superior regarding its sensitivity and suitability for high throughput analyses [13], are used to follow up and confirm seropositive cases.

The introduction of LPAIV in Tunisia in December 2009 has led to the spread of the disease in many parts of the country. Up to this date, there was no evidence on the ecology and the natural history of AIV transmission in Tunisia bearing in mind that most AIV outbreaks in humans and birds remain unpredictable and difficult to control. Besides, H9N2 subtype was reported to cause infection in humans [10] and public health officials, worldwide, are concerned about AI epidemics because of its potential risk to cross species barriers.

This report is the first study conducted on AIV infection in chickens and turkeys in Tunisia. It summarizes the findings of a national survey realized from October 2010 to May 2011 with regard to influenza virus infections, phylogenetic characteristics, proportion of infected flocks and associated risk factors.

## Materials and Methods

### Poultry flocks sampling and questionnaire administration

A cross sectional sero-epidemiological survey was assessed to estimate the flock-prevalence of influenza A virus infection was approved by the ministry of agriculture as a control and surveillance study during the period from October 2010 to May 2011.

This study was carried out on commercial flocks reared in 20 governorates from northern, central and southern Tunisia. A total of 800 flocks consisting of 187 layer, 453 broiler, 58 breeder broiler and 102 turkey flocks were enrolled in this study. The majority of sampled flocks were recorded as having respiratory symptoms, mortality or drop in egg production. None of these flocks received any influenza A virus vaccines. Layer flocks were of varying ages, ranging from 3 to 83 weeks old (mean age 45), breeder broiler were from 10–64 weeks old (mean age 37), the broiler flocks aged from 23 to 62 days of age (mean age 33) and turkeys were from 4 to 18 weeks (mean age 11). Twenty blood samples were separately collected from birds in each of the 800 flocks, centrifuged and stored at −20°C for further serological analyses. This should allow detection of AIV antibodies for a within-flock seroprevalence of 15% (5% type I error) and estimation of the between-flock prevalence with 3% precision at the 95% confidence level (expected prevalence was set at 50%, the value for which the sample size required is the largest) [14]. In addition, cloacal and tracheal swabs were also taken on 20 birds per flock. Given financial constraints, analysis was finally limited to the samples coming from only 400 of the 800 flocks.

All chicken sampling protocols were approved prior to the beginning of the study by the biological animal security committee of National Agriculture Ministry, and conducted by trained veterinarians. Simultaneously, a more detailed questionnaire was filled out to collect valuable information on the farm (poultry species, premises, close environment, biosecurity, etc) and the reared birds (age, health statue, vaccination program…). The owners agreed to participate in such study and a written informed consent document was obtained from each participating farm.

### Competitive ELISA assay (cELISA)

Serological evidence of avian influenza virus was detected using a Competitive Enzyme-Linked Immunosorbent Assay (cELISA) (ID-screen® Influenza A Antibody Competition ELISA Kit, ID-Vet, Montpellier, France) specific to influenza A nucleoprotein (NP) as described by Zhou et al [15]. The results were expressed as the percentage of competition value according to the following formula: (OD samples/OD negative)×100. Poultry flocks were considered positive for antibodies to influenza A NP if the average percentage of competition (mean value of 20 tested sera) is lower than 45.

#### Statistical analyses

Statistical analyses were carried out with the R software and the flock was considered as an epidemiological unit. Between-flock prevalence and confidence intervals were estimated using the epiR package.

Among the available data collected in the questionnaire, seven categorical variables were selected to study the risk of AI infection such as: area (geographical location of the farms), bird species, nearby poultry farms – including backyard – within a radius of 500 m around the farmhouse, nearby humid zone (proximity to river, lakes, swamps), biosecurity measures (building infrastructure, equipments, farm management), owner/workers circulating between farms and contacts with migratory birds (wild birds observed or not in the neighboring areas). These variables were first entered in univariate logistic regression models, with flock status (infected/not infected) as dependent variable. An initial multivariate model was built based on all the predictors with p≤0.25 (LR test) in the univariate screening. Variable selection for the final model was carried out through a backward elimination process based on the log–likelihood ratio test as recommended by Hosmer and Lemeshow (2000) [16]. Spatial autocorrelation between flocks was taken into account by including governorate as a random effect in the regression models. Between-flock seroprevalence was mapped using a geographical information system software (ArcGIS v.9.2^©^ ESRI Inc.).

To ascertain the effect of age upon the susceptibility to influenza virus infection, univariate logistic regression was also performed for long cycle poultry systems (layers, broiler breeders, and turkeys). Age was categorized into three categories based on the laying phase (before 18 weeks, from 18 to 32 weeks, after 32 weeks).

### Virus propagation and titration

Pooled swab (cloacal and tracheal) samples were propaged in 10-day-old SPF embryonated chicken eggs (ECEs) (Lohmann Ltz, Germany). Infective amino-allontoic fluids (AAF) were harvested at 96 h post incubation, tested for the presence of hemagglutinin (HA) activity [17], and titrated to determine the 50% tissue culture infective dose (TCID_50_)/ml, using primary chicken embryo fibroblast cultures (CEFs).

### Antigen enzyme immunoassay (Ag EIA)

An initial screening for AIV was done by AgEIA as described (ID-screen® Influenza A Antigen Capture, ID-Vet, Montpellier, France). In brief, the diluted swabs was mixed well and incubated (50 µl/well) into a microwell coated with Influenza A (NP) specific monoclonal antibodies for 25 min at room temperature. IgG conjugated with horse radish peroxidase was then added (100 µl/well) followed by color development with chromogen and substrate. The Cut-off level was calculated as three standard deviations above the mean optical density value of negative controls (threshold = mean OD_NC_±3SD). Pooled swab samples having OD value above the threshold level were considered positive.

### CEF cell culture

Primary chick embryo fibroblast (CEF) cells were cultured from SPF embryonated chicken eggs (Lohmann Ltz, Germany) (density of 1×10^4^ cells per well) in Dulbecco's Modified Eagle Medium (DMEM) (Gibco®, Invitrogen Life Technologies, Carlsbad, CA) containing 10% fetal bovine serum (FBS), 100 IU/ml penicillin G, and 100 µl/ml streptomycin. The 80% confluency reached cells were inoculated with 100 µl of AgEIA positive samples, at a multiplicity of infection (MOI) of 1∶1 in DMEM with 5% FBS supplemented with 2 µg/ml tosyl-phenylalanyl chloromethyl ketone (TPCK)-Trypsin (Gibco®) [18]. The supernatants were 10-fold diluted to calculate the infectious virus titer by determining the 50% tissue culture infective dose (TCID_50_) using the Reed and Muench method.

### Hemagglutination-inhibition (HI) test

An HI test was carried out using reference influenza A virus sera (kindly provided by FAO) of known anti hemagglutinin (HA) antibody (H5, H7, H9) titers as recommended in standard protocol [17]. The HI titer was expressed as the reciprocal of the highest serum dilution that completely inhibits hemagglutination. Serum samples indicating <8 HI titer were regarded as negative.

### RNA extraction

Viral RNA extraction was carried out using TRIzol® (Invitrogen, Carlsbad, CA, USA) as recommended by the manufacturer. The concentration and the purity of the extracted total RNA were determined by measuring the absorbance ratio at wavelength 260 nm over 280 nm using a spectrophotometer.

### Identification of Influenza A viruses

#### Real time RT-PCR (rRT-PCR) for IA typing and subtyping

Ag EIA result's confirmation was done by a real-time reverse transcriptase polymerase chain reaction (rRT-PCR) using One-Step Qiagen RT-PCR kit (Qiagen, Hilden, Germany) with primers and probes (Table S1) targeting the conserved region of the matrix (M) gene [19]. AIV positive samples were tested for H5, H7 [19] and H9 [20] subtypes following the described protocols. Samples with threshold (Ct) values <35 were considered positive.

### Conventional RT-PCR

The amplification of H9 and N2 genes was carried out separately with primers published by Ji [21] and Kwon [22], respectively. PCR products were run on 2% agarose gel, stained with ethidium bromide and visualized by UV illumination. The product was purified and extracted from the agarose gel with the Gel Kit (GenClean® II kit, North America, Solon Ohio). The DNA was quantified by NanoDrop® ND-1000 Spectrophotometer (NanoDrop Technologies, Inc., Wilmington, DE, USA) and five 10-fold dilutions of the DNA were performed and tested by real-time RT-PCR.

### Viral HA and NA gene's sequencing and phylogenetic analysis

The DNA templates were purified and sequenced by the Big Dye Terminator® v1.1 sequencing kit (Applied Biosystems, CA, USA). Post sequencing products were purified prior to running on 3730 XL DNA (ABI Prism 377, DNA sequencer, Applied Biosystem Inc., CA, USA) Analyzer.

Sequences were aligned using the Bioedit 5.0.6 program. The two segments were phylogenetically analyzed on the basis of their protein sequences. The MEGA5.01 version 3.65 program was used for tree building using the neighbor-joining method. The number of bootstrap replications was set to 1000. All branches supported by more than 50% bootstrap values were considered to be in the same group in the trees. Abbreviations used and GenBank accession numbers of different H9N2 viruses included in the phylogenetic analysis are listed in supplementary Table S2.

### Accession numbers

The GenBank sequence accession numbers for the two segments HA and NA of the four isolates included in this study are JQ952588 through JQ952592.

## Results

### Clinical signs

Flock records and survey sheets describing the clinical signs observed in the visited farms indicated a mortality rate of 10% to 30% with moderate to severe respiratory symptoms in broiler flocks. Infected layer, broiler breeder and turkey flocks showed moderate respiratory symptoms and severe drop in egg production.

### Seroprevalence and risk factor analysis

**Figure 1:**
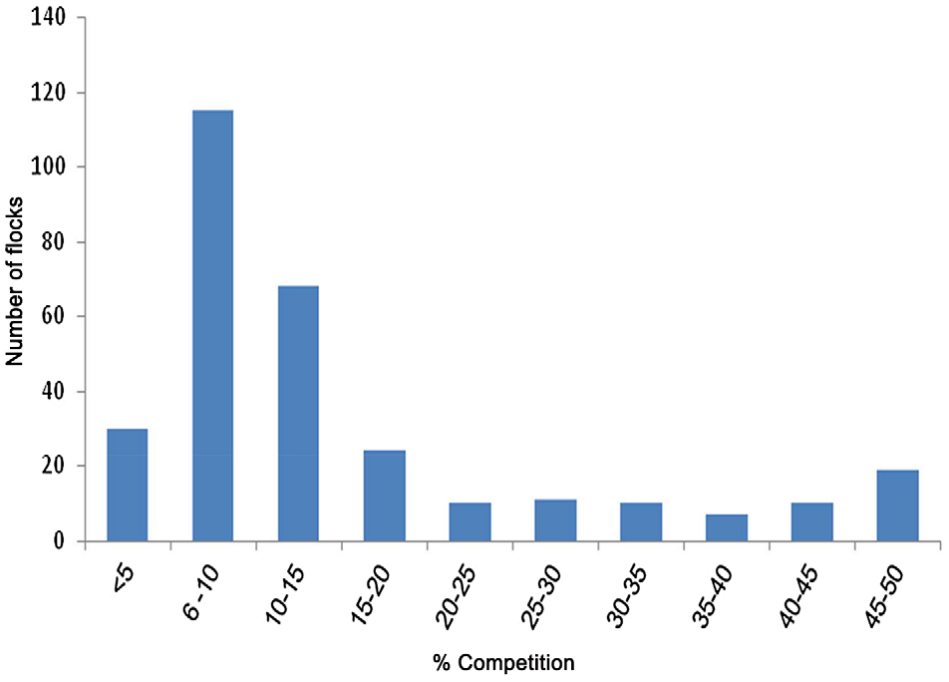
cELISA results for detection of antibodies to influenza A virus NP in commercial chicken and turkey sera. (Result was defined as positive when % of competition is ≤45%, doubtful at 45–50% and negative ≥50% competition).

**Figure 2:**
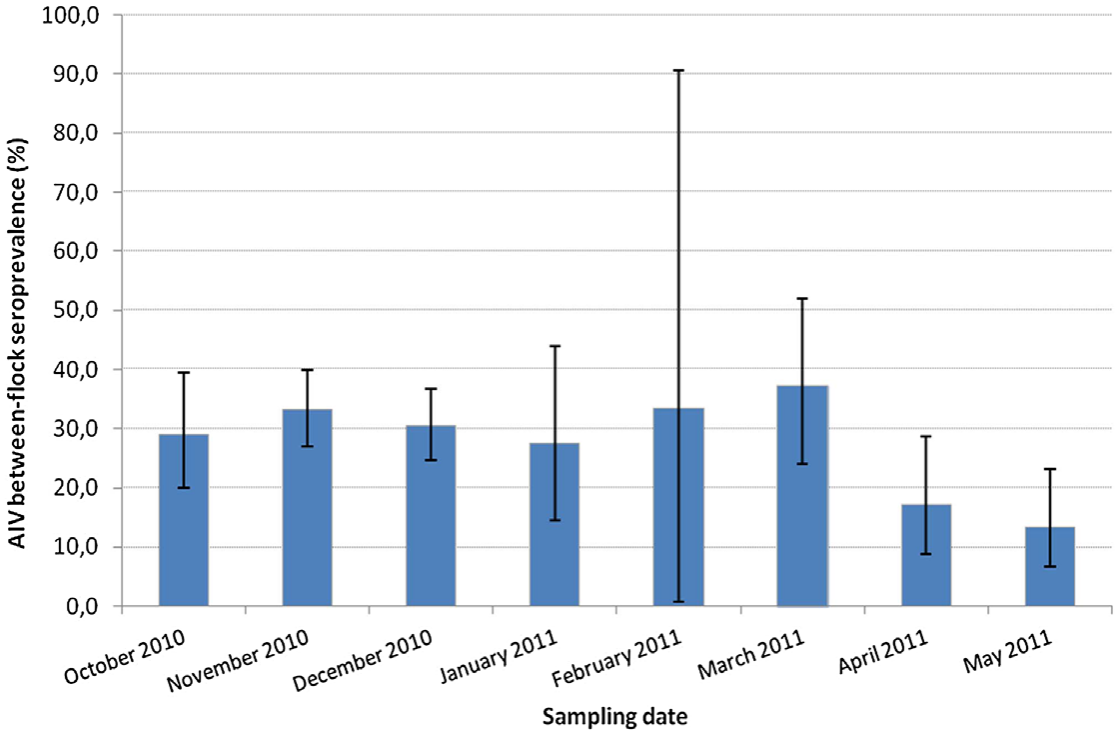
The monthly distribution of AIV between-flock seroprevalence (%) in commercial poultry flocks Tunisia (778 chicken and turkey flocks sampled from October 2010 to May 2011).

**Figure 3:**
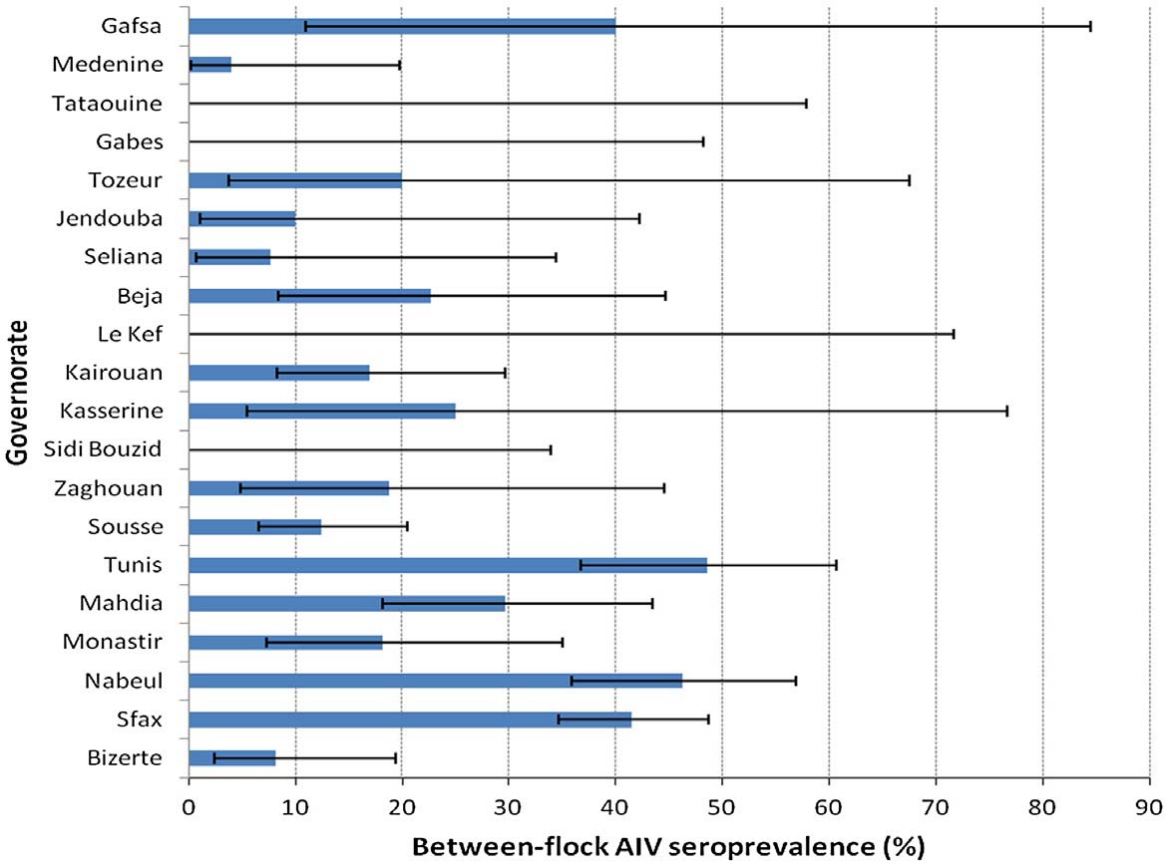
The between-flock AIV seroprevalence (mean proportion of poultry flocks positive regarding antibodies to AIV) in Tunisian governorates from October 2010 to May 2011.

Out of the 800 chicken and turkey flocks tested by competitive cELISA, 223 showed positive anti-NP antibodies. Figure 1 indicated positive sera peak at about 6–10% competition and 3% were considered doubtful. The overall between-flock seroprevalence was 28.7% (95% confidence interval, CI, 25.6–32.1). The highest levels of seroprevalence were observed during autumn and winter with a peaked in March at 37.2% (CI 24.1–51.9) (Figure 2). Highest seroprevalences were observed in Tunis (47.9%, CI 36.1–59.9), Nabeul (45.7%, CI 35.4–56.3) and Sfax (41.3%, CI 34.4–48.4) governorates where the majority of commercial farms are located (Figure 3).

**Figure 4:**
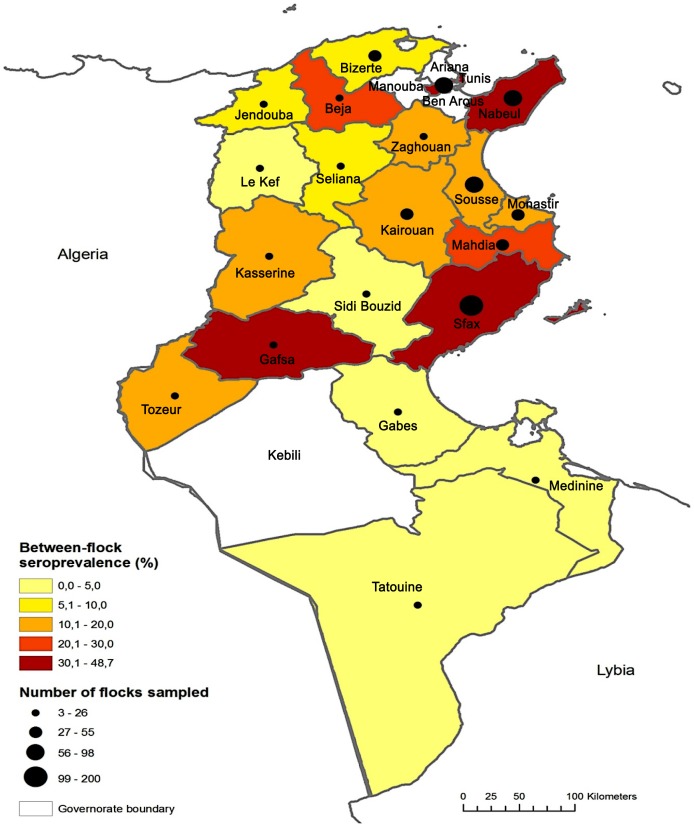
Map of the estimated between-flock AIV seroprevalence and number of commercial poultry flocks sampled by Tunisian governorates from October 2010 to May 2011.

Of the 7 candidate risk factors, 5 were significantly associated with avian influenza at p<0.25 (Table 1) and were selected for inclusion in the multivariate modeling process. Four variables were found significantly associated with the risk of AIV infection in the final model (Table 2). It appeared that between-flock seroprevalence is significantly higher (p<0.05) in farms on the coast including Bizerte, Tunis, Nabeul, Sousse, Mahdia, Monastir and Sfax as compared to the inland farms (Figure 4; Table 1).

**Table 1:** Results of the logistic regression screening of categorical risk factors associated with cELISA IA seropositivity in 624 commercial poultry flocks during the 2010–2011 outbreaks in Tunisia.

Variables	Number of flocks	OR	CI 95%	Wald's test p-value
**geographical area**				
inland	157	ref		
coastal	467	3.75	2.24–6.28	<0,001
**birds species**				
broiler	339	ref		
layer	150	4.98	3.25–7.63	<0,001
broiler breeder	53	4.01	2.28–7.38	<0,001
turkey	82	1.56	0.88–2.79	0.13
**nearby poultry farms**				
no	329	ref		
yes	295	1.41	0.99–2	0.05
**nearby humid zone**				
no	522	ref		
yes	102	1.11	0.7–1.77	0.6
**Owner/workers circulating between farms**				
no	519	ref		
yes	105	0.66	0.4–1.09	0.10
**biosecurity measures**				
good	321	ref		
low	303	1.74	1.23–2.48	0.002
**contact with migratory bird**				
no	608	ref		
yes	16	1.14	0.39–3.34	0,81

**Table 2:** Results of the final multivariate analysis (logistic regression with governorate as a random effect) of risk factors associated with the serological status of commercial poultry flocks regarding avian influenza virus (AIV) in Tunisia during the 2010–2011 outbreaks.

Variables	Number of flocks	OR	CI	p
**geographical area**				
inland	157	ref		
coastal	467	3.51	1.32–9.31	0.01
**bird species**				
broiler	339	ref		
layer	150	3.94	2.41–6.43	<0.001
broiler breeder	53	3.72	1.85–7.46	<0.001
turkey	82	0.96	0.51–1.80	0.89
**nearby poultry farms**				
no	329	ref		
yes	295	1.45	0.94–2.23	0.09
**biosecurity measures**				
good	321	ref		
low	303	1.57	1.01–2.43	0.05

Interestingly, seroprevalence in broiler flocks (17.6%, CI 14.2–21.5) was also significantly lower than that seen in layers (50.3%, CI 42.6–57.4) and broiler breeders (46.5%, CI 33.3–60.1) (Table 2).The risk of AIV infection was significantly higher for commercial farms with low biosecurity level (sanitary and management failures) as compared to those showing medium to high biosecurity measures (better sanitary and management measures applied) (Table 1).

To a lesser extent (p = 0.09), the risk of AIV was found associated with the presence of a poultry farm in a 500-m radius.

The influenza virus infection was less prevalent during young age (<18 weeks) than during the laying phase and after for layers and breeder broilers (Table 3).

**Table 3:** Seroprevalence in different age group of seropositive flocks.

Flocks	Age	Seroprevalence % (No/total)	Odds ratio (OR) (95% CI)	p-value
**Layers**	>32 w	47.2 (56/106)	Ref	
	18–32 w	56.6 (17/30)	1.17 (0.52–2.64)	0.71
	<18 w	15.4 (2/13)	0.16 (0.03–0.77)	0.02
**Breeder broilers**	>32 w	46.2 (12/26)	Ref	
	18–32 w	64.7 (11/17)	2.14 (0.61–7.53)	0.24
	<18 w	10.0 (1/10)	0.16 (0.03–0.77)	0.07
**Turkeys**	>11 w	20.0 (9/45)	Ref	
	≤11w	29.7 (11/37)	1.69 (0.61–4.67)	0.31

w: weeks, Ref: reference.

### Virological analyses

**Figure 5:**
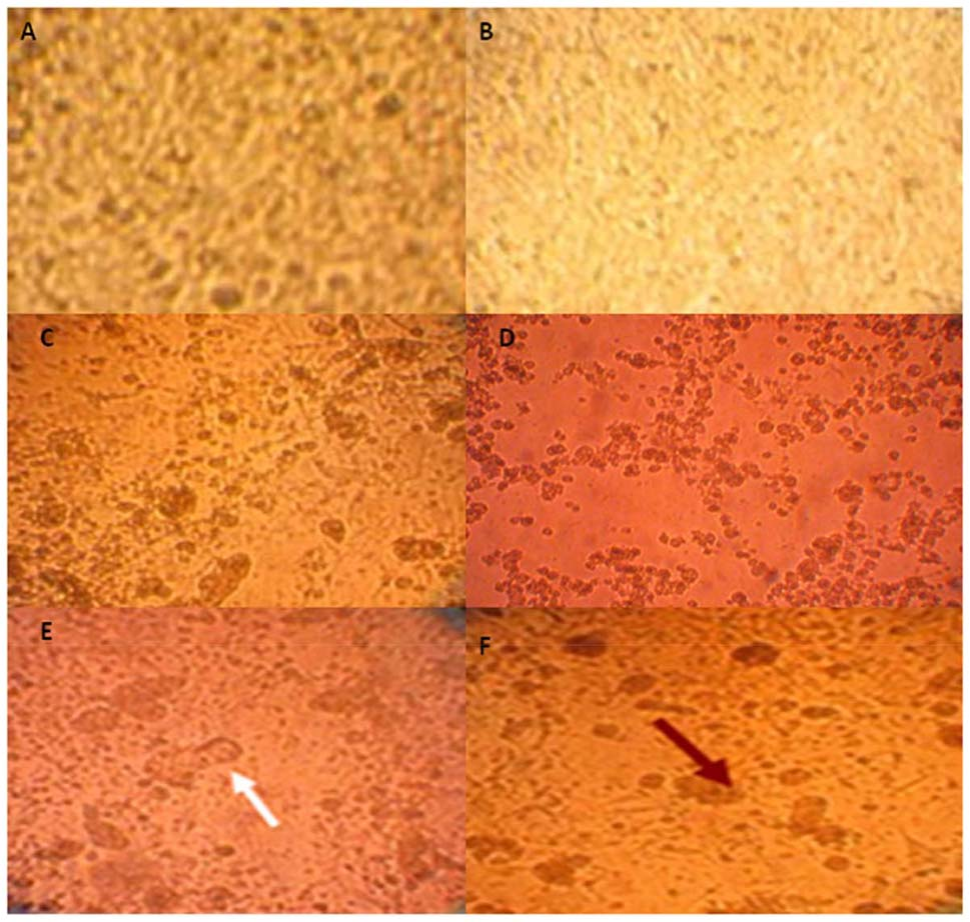
Pathogenicity of CEF cells to avian H9N2 influenza virus in the presence (CDEF) and absence (B) of trypsin. (A) mock cells. Arrows showed syncytia and plaque formations.

Out of the 400 tested flocks, 40 (10%) were positive by Ag ELISA for NP antigen and by rRT-PCR for M gene (ct value in the range of 15–33). Among them, only 29 samples showed cytopathic effect in chicken embryo cell (CEC) cultures, characterized by plaque formation only in the presence of trypsin (Figure 5). Chicken embryos inoculated with the selected samples died within 24–72 hr in only 20 cases. The HA assays revealed that the HA titers ranged from 5 to 9 log_2_. HA subtyping was done by HI test using H5, H7, and H9 specific antisera, for 20 out of the 40 positive samples. They were all identified as H9 subtype with HI titer ranging from 4 to 8 log_2_. None of them were identified as H5 and H7 subtypes. Besides, all rRT-PCR positive samples for gene M detection were also identified as H9 subtype by the same specific assay.

The specificity of the primers was examined by rRT-PCR, using template extracted from H5N1, H7N3, H1N1 and other avian viruses, including Newcastle disease, infectious bronchitis and infectious bursal disease viruses. None of the above viruses showed a positive result, indicating that primers used were indeed highly specific to avian influenza A virus and H9 subtype.

**Figure 6:**
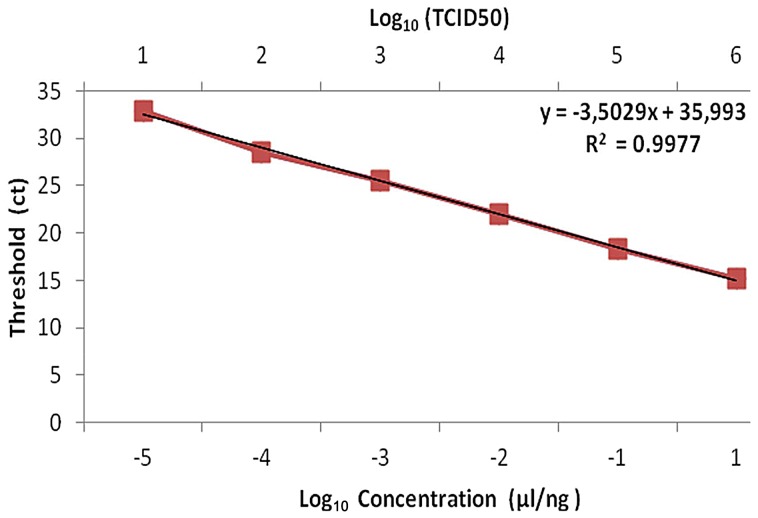
A linear relationship between threshold cycle and serially diluted DNA concentration or TCID50 values. PCR efficiency ((10^3.503^−1)×100) was 99.87% as indicated by the slope (m = −3.503). The standard curve was generated from amplification of the H9 gene with each point represents the mean of the results from three determinations.

The detection and the quantification of AI viral titer were performed by rRT-PCR using various amounts of purified DNA (3.7 ng/µl) or TCID50 values (10^6^ TCID50/ml). As shown in Figure 6, the analysis of threshold cycle (Ct) signals as function of log10 concentrations and TCID_50_ titers showed a nearly linear decrease of Ct value with increased virus titer. The detection limit of rRT-PCR was 3.7×10^−5^ ng/µl (while by RT-PCR, the limit was 3.7×10^−2^ ng/µl) or 1 TCID_50_/ml, respectively. The correlation coefficient R^2^ value was 0.99, indicating linear regression between the standard curve line and the individual Ct data points.

It seems that the confirmation of Ag EIA positive results was high by rRT-PCR, but low as compared to Cell culture and egg inoculation.

In addition, conventional RT-PCR detected a 1500 bp and a 410 bp products specific to the H9 and N2 genes respectively.

Four isolates from areas with the highest seroprevalence (Nabeul, Sfax and Tunis) were selected for HA and NA gene sequencing to discuss molecular evolution during H9N2 AIV outbreaks. Mixed speculations were chosen: broiler, layer, broiler breeder and turkey within the AIV isolates correspond to several seasons.

**Figure 7:**
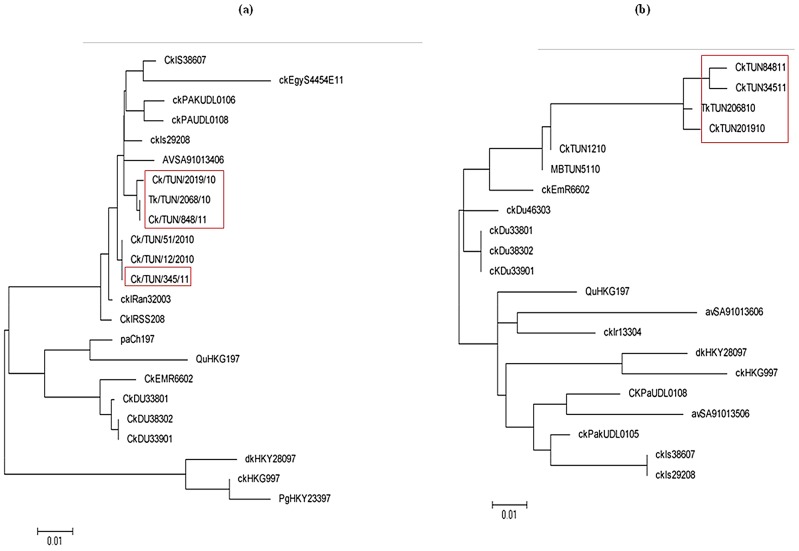
Phylogenetic relationships of the HA (a) and NA (b) genes of 2010–2011 Tunisian strains (H9N2). Horizontal distances are proportional to the minimum number of nucleotide differences required to join nodes and sequences. Vertical distances are for spacing branches and labels. The phylogenetic trees were generated by using the neighbor-joining algorithm assessed by bootstrap analysis with 1,000 replications. Abbreviations used in virus designation are as follows: Av, avian; Ck, chicken; Dk, duck; gs, goose; Qu, quail; Tk, Turkey; Pa, parakeet. The in box strains are avian H9N2 Influenza viruses' isolates that were sequenced in the present study.

Phylogenetic analysis of HA (Figure 7a) and NA (Figure 7b) sequences revealed that the four isolates belonged to the Eurasian clade and fell within the same G1-like lineage. All isolates identified during 2011 and 2010 were most closely related (similarity out of 96%) to the A/Migratory Bird/TUN/51/2010 and A/Ck/TUN/12/2010 previously isolated.

Multiple alignment of the HA gene (Table 4) revealed that these strains have the same P-A-R-S-S-R ↓ G amino acid motif at the cleavage site and carried the aa substitution Q234L (H9 numbering) that correlated with a shift in affinity to human type SA α2,6 receptor type. The current isolates maintained conserved residues at the receptor-binding pocket: P116, T163, H191, A189 and I235.

**Table 4:** Analysis of amino acid sequences of the HA and NA proteins of current Tunisian isolates.

Starins	HA															NA												
	Receptor binding site				aa residue at position				Connecting peptide					glycosylation site		Hemadsorption site									Framework site			
	191	198	202	203	233	234	253	265						166	183	356	360	368	370	372	378	384	400	403	432	442	444	451
**CK/TUN/2019/10**	H	A	L	Y	G	L	I	F	P	R	R	S	R	+	+	D	A	K	L	A	R	N	S	N	K	H	**V**	**G**
**Tk/TUN/2068/10**	.	.	.	.	.	.	.	.	.	.	.	.	.	+	−	.	.	.	.	.	.	.	.	.	.	.	.	.
**CK/TUN/345/11**	.	.	.	.	.		V	Y	.	.	.	.	.	+	−	.	.	.	.	.	.	.	R	D	.	N	I	.
**CK/TUN/848/11**	.	.	.	.	.	.	.	.	.	.	.	.	.	+	−	.	.	.	.	.	.		R	D	.	.	.	.
**MB/TUN/51/10**	.	.	.	.	.	Q	V	Y	.	.	.	.	.	+	−	N	V	.	.	.	.	.	T		Q	N	I	R
**CK/TUN/12/10**	.	.	.	.	.		V	Y	.	.	.	.	.	+	−	N	V	.	.	.	.	.	N		Q	N	I	.
**AV/SA/910136**	.	T	.	.	.		V		.	.	.	.	.	−	−	T		E	S	V			T		Q	N	I	.
**CK/Is/386/07**	.	E	.	.	.	.	V	.	.	.	.	.	.	−	−	N	V		S		K		N		Q	N	I	.
**CK/HK/G9/97**	.	.	.	.	D	Q	V	Y	.	.	.	.	.	−	−	T	.	.	S	S	.	.	T			N	I	.
**SW/HK/562/05 (H1N2) (CY086796)**																.	.	S	.	.	.	.	T	S	Q	N	I	.
**CK/NY/Sg-00301/97 (H2N2) (CY054349)**																Y	V	.	.	.	.	.	T	N	Q	N	.	.
**Md/Qub/11033/06 (H3N2) (CY095396)**																N	V	.	.	.	.	A	.	N	Q	N	.	.
**Md/Mary/792/02 (H5N2) (GU053507)**																Y	V	.	.	.	.	.	T	N	Q	N	.	.
**CK/Calif/1316/01 (H6N2) (AF474044)**																S	V	.	.	.	K	A		N	Q	N	.	.
**Ph/Sh/7503/04 (H6N2) (EU050152)**																.	.	.	.	.	.	.	S	R	Q	N	.	.
**Ck/NY/4447-7/94 (H7N2) (AY254226)**																N	V	.	.	.	A	N	.	.	Q	N	.	.

The N-linked glycosylation sites are conserved at positions 82, 105, 141, 218, 298, and 305 in the HA1. Additional glycosylation site was found at aa position 166 in all Tunisian isolates expect the CK/TUN/2019/10 H9N2 strain which carries another glycosylation at residue 183 (Table 4).

The CK/TUN/345/11 H9N2 variant conserved V and Y at position 253 and 265, respectively compared with the previously identified strains MB/TUN/51/10 and CK/TUN/12/10, while the three other variants carried V253I and Y265F substitutions.

The NA genes of the 2010–2011 isolates differed from previously identified Tunisian strains by six aa substitutions: N356D, V360A, T384N, Q432K and S442H, I444V (except CK/TUN/345/11 variant). In addition, the two 2011-strains exhibited three new mutations S400R, N402D and G451R.

The framework and the hemadsorption sites of the NA contain R^371^, A^372^, N^402^, E^425^ and K367, L370, A372, D401, Y406 respectively.

## Discussion

The current study attempted to monitor AIV in commercial poultry flocks and investigate possible risk factors associated with AI seroprevalence at the farm level. The poultry industry, composed of broilers, layers, broiler breeders and turkeys, is one of the most developed animal sectors in Tunisia. In 2008, the number of commercial farms has been estimated at 3.000, with 84.000 poultry. Poultry meat production, estimated to be 160.000 tons in 2010 represented 56% of the total meat production in Tunisia. The traditional sector, representing 2.9 millions of laying hens and 1.4 millions of chickens, also contributes to poultry production and food security in the country [23]. In 2006, rumors following the detection of highly pathogenic avian influenza in poultry farms on the African continent had an economic impact of 19 million dollars on the Tunisian poultry industry [23].

The national surveillance network of myxoviruses (Avian Influenza and Newcastle Disease Viruses) has been set up since 2006 and has been very successful. It has contributed to the early detection of the first introduction of AI in Tunisia in December 2009, and allowed the follow up of its evolution, from the north to the center and the south of the country, especially in layer flocks [24].

The study showed some limitations that should be taken into account before conclusions are drawn. First, seroprevalences should be regarded cautiously as the 800 flocks enrolled in the present study may have not been chosen randomly among all Tunisian commercial poultry farms. Indeed, questionnaire showed that most of the farms enrolled reported clinical signs. This study may thus not depict the overall AIV situation in Tunisia, but certainly provides a precise description of the AIV seroprevalence in Tunisian flocks showing clinical signs. Second, the number of poultry samples collected in the field varied greatly according to the month of year and the governorates. This makes the interpretation of geographical and temporal variations difficult, due to the heterogeneity of seroprevalence confidence intervals. Also, the temporal pattern of seroprevalence should be considered in regard to the persistence of AIV antibodies in poultry over the course of time. Further longitudinal studies, based on random sampling of farms and birds and carried out over longer periods of time, are desirable to address these limitations. At least, the questionnaire survey resulted in missing information for 176 of 800 farms. These farms were excluded from the statistical risk factor analysis, but the influence of missing values on the robustness of results could not be deeply analyzed. Despite these limitations, this study brings new insights to the epidemiology of avian influenza in the Maghreb and Mediterranean countries.

The overall between-flock seroprevalence of AIV antibodies was 28.7% (CI 25.6–32.1), which demonstrates that AIV was present in Tunisia during 2010 to 2011. The cELISA, was assessed as sensitive, specific (100%, respectively as provided) versus (95% and 96% in chickens, 86% and 88% in ducks, 97% and 100% in Turkeys, 87% and 100% in goose, and 91% and 97% in swine) and reliable tool for the study of AIV epidemiology [25]. Besides, previous studies suggested that cELISAs should be effective for a large-scale surveillance of AIV in avian and other species [26]. A higher AIV seroprevalence (71,3% ) was reported in adults birds by Toennessen et al. [27]. Interestingly, our finding is consistent with the high flock-seroprevalence recorded previously on chicken flocks experiencing respiratory signs or mortality in Jordan [28] and Egypt [29].

We found that high seroprevalences were observed in the coastal areas, where most commercial farms are located, compared to the inner regions of the country. This result may reflect an intense low pathogenic AI circulation in areas with high densities of poultry farms and slaughterhouses, as it has previously been described for the highly pathogenic H5N1 virus [30].

In this report, we also evidenced that AIV infection may be maintained over several consecutive months in Tunisian commercial flocks. Additional longitudinal prevalence studies would be necessary to examine temporal variations of AIV infection,, but our results nevertheless suggest that AIV circulation may be more important throughout autumn and winter than in spring. The temporal fluctuations of AIV seroprevalence in this study probably reflect different proportions of poultry types sampled from one month to another. Indeed, broiler flocks – which showed lower risk of AIV seropositivity than layers, breeders or turkeys – represented the majority (more than 75%) of the flocks sampled in April and May, when seroprevalence was low (<20%). However this may not be the only explanation for monthly variations as seroprevalence remained over 25% in January and March, with broilers representing 64% and 62% of the flocks sampled at that period of time. It has also been suggested that AIV prevalence may vary with contacts between poultry and wild birds and virus survival under environmental conditions, including temperature [31]. At last, between-flock seroprevalence might also be influenced by the seasonality of poultry production and trade in Tunisia.

Our study showed higher prevalences for layer (50.3%) and broiler breeder flocks (46.5%) compared to broiler chickens. Recent report also suggested that broiler flocks were less affected with H9: only 1/9 (11.1%) flocks were found seropositive to H9 versus 13/18 (72.2%) and 7/12 (58.3%) for H9, in the examined layer and breeder poultry farms, respectively [32]. In fact, the long life span of layers and broiler breeders allows the development of a stronger immune response, as a result of the longer exposure to the risk of AI infection. This phenomena might explain the higher prevalence detected by cELISA as compared to broilers, which have a short life span and for which the blood samples may have been collected at the acute stage of infection. Results suggest that for the layer flocks, the AIV infection may have occurred during the laying phase and increased after. These findings may help to explain the observed drop in egg production.

Interestingly, a large proportion of flocks more than 4 weeks old and unvaccinated were found to be infected with AIV. This suggests a direct exposure of Tunisian commercial flocks to AI. Similar finding found that 5 weeks old chicks were more likely to have produced antibodies in response to AIV infection than those of 3 week old [33].

In addition, our survey showed that the biosecurity level of poultry farms was significantly associated with the risk of AIV infection. Commercial poultry sector in Tunisia includes various farm management conditions with low-biosecurity level (wooden or open poultry premises, products sold on local markets), which presents high risk regarding AIV infection (OR = 1.57, CI 95%: 1–2.4), to higher-biosecurity level observed in many industrial farms with birds kept in a closed house, maintaining highly restricted physical barriers and applying strict disinfection and hygienic procedures (elimination of infected flocks to limit possible AIV spread). Besides, the poultry producers seek assistance for diseases diagnosis through professionals and dedicated trained personnel and reinforcement of biosecurity measures.

No association was found in our work between the risk of AIV and the possible contact with migratory birds, as well as with owner/workers circulating between farms. However, the increased number of seropositives in farms located within the migratory route of wild birds was approved in previous study [28], [34]. More comprehensive questionnaires and studies should be done to better elucidate the role of these factors.

The screening for the AIV infections in flocks was performed by Ag ELISA and confirmed by rRT-PCR using M primers allowing detection of AIVs type A.

Tissue cultures (CEF) were used as another indicator for the presence of the virus. None of the H9N2 isolates tested showed cytopathic effect (CPE) in infected CEF monolayer in the absence of trypsin whereas CPE could be observed by 72 h post infection in presence of trypsin, in 29 out of 40 samples. This clearly indicated that the Tunisian H9N2 isolates are of low pathogenicity, which is further supported by sequencing data results.

Our findings also demonstrated that Ag EIA has higher sensitivity than virus detection through egg and cell culture inoculations and underlined its potential and value in IA routine diagnostic.

Positive cases were then subtyped by both HI test using specific H9 polyclonal antisera and rRT-PCR assay followed by conventional RT-PCR using subtype-specific primers for the genes H9 and N2, respectively. The rRT-PCR assay for M and H9 genes revealed significantly higher numbers of positive samples, and confirmed all the positive results revealed by AgELISA. All new strains were subtyped as H9N2 by real time and conventional RT-PCR

The standard curve was generated from Ct values plotted against viral titers expressed as diluted H9 DNA concentrations or as 50% tissue culture infective doses (TCID_50_)/ml. Interestingly, rRT-PCR test, which can rapidly identify type A influenza as well as subtype H9, was very useful for the screening of AIV. It can also be a very valuable tool for the control of the disease transmission in poultry and in humans and can help reducing economic losses associated with AIV outbreaks in poultry.

Real time RT-PCR assay offered several advantages over conventional RT-PCR and other virological detection assays such as Ag EIA, hemagglutination inhibition or cell cultures. In addition to the relatively short run time, rRT-PCR is a quantitative assay and can be used for absolute or relative viral RNA quantified. This TaqMan- probe-based RT-PCR assay targeted the M and the HA genes of AIV. It has been reported that the rRT-PCR, used for the detection of human and avian influenza viruses, is very sensitive (2 copies of *in vitro*-transcribed RNA or 0.05 TCID_50_ per reaction) and highly specific [20].

Phylogenetic analyses based on the HA and NA genes revealed that current Tunisian (H9N2) variants are closely related to each other and to Middle Eastern strains with a similarity score of up to 96% within the members of the G1 lineage. Interestingly, 2010–2011 Tunisian H9N2 strains belong closely to the same clade as the MB/TUN/51/2010 H9N2 strain previously isolated from migratory bird [24]. In fact, our surveillance study was carry out during the seasonal coming of migratory bird and phylogenetic analysis may underline the role of wild birds in the introducing of the virus. It has been suggested that the migratory birds play an important role in the virus introduction but that the spread of AIV to other wild and domestic species, present in their migratory pathways may be limited [29], [34]. Environmental persistence of the virus in various aquatic habitats (lakes, rivers, ponds) of wild bird population may play a key role in its transmission within animal population and its persistence in the environment [35]. Previous study showed that H9N2 viruses are transmitted by the tracheal route via aerosol mode and a lesser extent by feces [36]. The spreading mode could be by the contact with wild and aquatic birds, leadding to an interspecies transmission and great variability in host-range restriction and genetic diversity [35], [37].

The cleavage site motif PARSSR*GLF consistent with the characterization of LPAI and the presence of Leu (L) at position 226 of all isolates indicated their binding potential with SA α 2,6 receptor [38]. New additional glycosylation was shown at aa residue 183 of the Ck/TUN/2019/10 isolate, but whether this change might affect the viral characteristics needs to be further explored.

Moreover, three specifically Tunisian variants carried new V253I and Y265F substitutions previously observed in Saudi Arabian, Israeli and Pakistani strains. The role of these substitutions should be examined.

NA gene was more polymorphic: drift mutations have occurred in the framework and the hemadsorption sites in the variants resulting in 9 substitutions. Three were non- synonymous substitutions. Three substitutions V360A, T384N, Q432K, not similar to the two Tunisian identified strains, were previously found in H9N2 reference strains (such as Saudi Arabia, Israel and G9 strains). Only current Tunisian isolates harbored three new mutations N356D, S442H and I444V similar to other N2 subtypes (H1N2, H6N2, H5N2, H7N2, H3N2 and H2N2). Interestingly, three S400R, N402D and G451R substitutions found exclusively in 2011 Tunisian strains. However, the biological significance of these mutations is not yet known.

Considering substitutions detected in the isolate from turkey, it is interesting to evaluate adaptative mutations in the new host. In summary, these drift mutations and the addition or deletion of a glycosylation site may reflect different evolutionary pressures applied to the two glycoproteins HA and NA.

A total of 120 out of 400 flocks that was negative in rRT-PCR assay, showed positive reactions for anti-NP antibodies in cELISA. This lower detection rate revealed by rRT-PCR may be related to the fact that AIV is present for a short period after initial infection and replicates poorly in the host, whereas the antibody responses last longer after infection.

For the 22 flocks revealed positive by rRT-PCR, cELISA was negative; this may suggest that the humoral response was not yet detectable.

## Conclusion

In conclusion, the current study showed evidence that AIV H9N2 strains are present in commercial different poultry species and regions of Tunisia, which highlights the potential risk of AIV infections.

Determining the subtype of the circulating AIV is very important to understand its evolutionary relationship with local as well as regional strains. Subsequently, amino acid substitutions in HA and NA proteins that are located at antigenic sites, require constant evaluation of the best possible vaccine candidates.

Strict enforcement of biosecurity measures and possible vaccination of all poultry flocks with continuous monitoring of poultry stations may ensure reduction of AIV prevalence and avoid emergence of more pathogenic strains.

## Supporting Information

Table S1 **Primers used for real time and conventional RT-PCR.** ^a^Codes for mixed bases positions: Y = C/T, W = A/T.
(DOC)

Table S2 **Abbreviations used and GenBank accession numbers for H9N2 Avian Influenza virus isolates included in phylogenetic analysis.** ^a^ Viruses whose HA and NA genes were sequenced in the present study; N.D, not done.
(DOC)
