# Supplementary material for: Risk Factors and Characteristics of Low Pathogenic Avian Influenza Virus Isolated from Commercial Poultry in Tunisia
Source: PLoS One. 2013 Jan 11;8(1):e53524. doi: 10.1371/journal.pone.0053524 (PMC3543454; doi:10.1371/journal.pone.0053524)
Supplement: Table S1 — Primers used for real time and conventional RT-PCR. aCodes for mixed bases positions: Y = C/T, W = A/T. (DOC) [file pone.0053524.s001.doc]

| **Target** | **Primer/ probe** | **Sequence (5’-3’)^a^** | **Reference** |
| --- | --- | --- | --- |
| **Influenza A type** | M+ 25 | AGA TGA GTC TTC TAA CCG AGG TCG | **19** |
|  | M- 124 | TGC AAA AAC ATC TTC AAG TCT CTG |  |
|  | M+ 64 | **FAM** TCA GGC CCC CTC AAA GCC GA **TAMRA** |  |
| **Avian H5** | H5LH1 | ACA TAT GAC TAC CCA CAR TAT TCA G | **19** |
|  | H5RH1 | AGA CCA GCT AYC ATG ATT GC |  |
|  | H5PRO | **FAM** TCW ACA GTG GCG AGT TCC CTA GCA **TAMRA** |  |
| **Avian H7** | LH6H7 | GGC CAG TAT TAG AAA CAA CAC CTA TGA | **19** |
|  | RH4H7 | GCC CCG AAG CTA AAC CAA AGT AT |  |
|  | H7PRO11 | **TAMRA** CCG CTG CTT AGT TTG ACT GGG TCA ATC T **BHQ** |  |
| **Avian H9** | H9F | ATG GGG TTT GCT GCC | **20** |
|  | H9R | TTA TAT ACA AAT GTT GCA Y CT G |  |
|  | H9PRO | **FAM** TTC TGG GCC ATG TCC AAT GG **TAMRA** |  |
| **Avian H9** | H9.1 | CCAAAGAATTGCTCCACACAGA | **21** |
|  | H9.2 | GCACAAGAGATGAGGCGACAGT |  |
| **N2** | N2.F | CTTGTTGGCGACACACCAAGRAA | **22** |
|  | N2.R | GAGCCTGTTCATAGGTACCTGA |  |
