# Supplementary material for: Risk Factors and Characteristics of Low Pathogenic Avian Influenza Virus Isolated from Commercial Poultry in Tunisia
Source: PLoS One. 2013 Jan 11;8(1):e53524. doi: 10.1371/journal.pone.0053524 (PMC3543454; doi:10.1371/journal.pone.0053524)
Supplement: Table S2 — Abbreviations used and GenBank accession numbers for H9N2 Avian Influenza virus isolates included in phylogenetic analysis. a Viruses whose HA and NA genes were sequenced in the present study; N.D, not done. (DOC) [file pone.0053524.s002.doc]

| **Reference strains** | **Abbreviations** | **Accession number** | |
| --- | --- | --- | --- |
|  |  | **HA** | **NA** |
| **Turkey/Tunisia/2019/10^a^** | Tk /TUN/2019/10 | JQ952588 | JQ952592 |
| **Chicken/Tunisia/2068/10^a^** | Ck /TUN/2068/10 | JQ952589 | JQ952593 |
| **Chicken/Tunisia/345/11^a^** | Ck /TUN/345/11 | JQ952590 | JQ952594 |
| **Chicken/Tunisia/848/11^a^** | Ck /TUN/848/11 | JQ952591 | JQ952595 |
| **Chicken/Tunisia/12/10** | Ck /TUN/12/10 | JF323006 | JF323008 |
| **Migratory bird/Tunisia/51/10** | MB/TUN/51/10 | JF323007 | JF323009 |
| **Quail/Hong Kong/G1/97** | QuHKG197 | AF156378 | AF156336 |
| **Duck/Hong Kong/Y280/97** | DkHKY28097 | AF156376 | AF156394 |
| **Chicken/Hong Kong/G9/97** | CkHKG997 | AF156373 | AF156391 |
| **Chicken/Pakistan/UDL-01/05** | CkPaUDL0105 | CY038458 | CY038412 |
| **Chicken/Pakistan/UDL-01/08** | CkPaUDL0108 | CY038410 | CY038460 |
| **Avian/Saudi Arabia/910135/06** | AvSA91013506 | GU050287 | GU050297 |
| **Avian/Saudi Arabia/910136/06** | AvSA91013606 | GU050295 | GU050289 |
| **Chicken/Israel/386/07** | CkIs38607 | FJ464618 | FJ464619 |
| **Chicken/Israel/292/08** | CkIs29208 | FJ464717 | FJ464616 |
| **Chicken/Emirates/R66/02 CY076723 CY076725** | CkEmR6602 | CY076723 | CY076725 |
| **Chicken/Dubai/338/01** | CkDu33801 | EF063520 | EF063524 |
| **Chicken/Dubai/339/01** | CkDu33901 | EF063514 | EF063521 |
| **Chicken/Dubai/383/02** | CkDu38302 | EF063515 | EF063522 |
| **Chicken/Dubai/463/03** | CkDu46303 | EF063516 | EF063523 |
| **Chicken/Egypt/ S4456B/11** | CkEgtS4456B11 | CY110928 | CY110926 |
